# Do Atypical Antipsychotics Have Antisuicidal Effects? A Hypothesis-Generating Overview

**DOI:** 10.3390/ijms17101700

**Published:** 2016-10-11

**Authors:** Maurizio Pompili, Ross J. Baldessarini, Alberto Forte, Denise Erbuto, Gianluca Serafini, Andrea Fiorillo, Mario Amore, Paolo Girardi

**Affiliations:** 1 Department of Neurosciences, Mental Health and Sensory Organs, Suicide Prevention Center, Sant’Andrea Hospital, Sapienza University of Rome, Rome 00189, Italy; alberto.forte@yahoo.it (A.F.); denise.erbuto@gmail.com (D.E.); paolo.girardi@uniroma1.it (P.G.); 2 International Consortium for Mood and Psychotic Disorders Research, Mailman Research Center, McLean Hospital, Belmont, MA 02468, USA; rbaldessarini@mclean.harvard.edu; 3 Department of Psychiatry, Harvard Medical School, Boston, MA 02115, USA; 4 Department of Neuroscience, Rehabilitation, Ophthalmology, Genetics, Maternal and Child Health, Section of Psychiatry, University of Genoa, Genoa 16126, Italy; gianluca.serafini@unige.it (G.S.); mario.amore@unige.it (M.A.); 5 Department of Psychiatry, University of Naples SUN, Naples I-80138, Italy; anfioril@tin.it

**Keywords:** antipsychotic, aripiprazole, asenapine, atypical, clozapine, olanzapine, quetiapine, risperidone, second-generation, suicide, ziprasidone

## Abstract

Modern antipsychotic drugs are employed increasingly in the treatment of mood disorders as well as psychoses, stimulating interest in their possible contributions to altering suicidal risk. Clozapine remains the only treatment with an FDA-recognized indication for reducing suicidal risk (in schizophrenia). We carried out a systematic, computerized search for reports of studies involving antipsychotic drug treatment and suicidal behaviors. A total of 19 reports provide data with preliminary support for potential suicide risk-reducing effects of olanzapine, quetiapine, ziprasidone, aripiprazole, and asenapine in addition to clozapine, and provide some support for antipsychotic drug treatment in general. These preliminary findings encourage further testing of antipsychotics for effects on suicidal behavior, making use of explicit, pre-planned assessments of suicidal behavior.

## 1. Introduction

Suicide is highly associated with psychiatric illnesses, particularly major affective and psychotic disorders. A clinically important insight arising over the past two decades is that suicidal behavior may be amenable to medicinal treatments, including lithium, perhaps less so with mood-stabilizing anticonvulsants, and possibly with antidepressants in adults more than juveniles [1,2]. Notably, too, the oldest atypical antipsychotic agent, clozapine (initially patented in 1960), is the only treatment with FDA recognition of beneficial effects against suicidal risk (in schizophrenia patients), as is discussed below. First-generation neuroleptic agents, though in wide clinical use since the 1950s, are not well evaluated for effects on suicidal behavior [3]. However, emerging epidemiological evidence suggests that they may reduce the risk of death by suicide or other causes in psychotic disorder patients, although probably less than clozapine [4,5,6]. Recognition of the antisuicidal effects of clozapine in schizophrenia encourages interest in the possibility that other modern antipsychotics may have such effects, and perhaps not be limited to schizophrenia. This possibility is suggested by accumulating evidence from controlled treatment trials that some modern antipsychotics have beneficial effects in major depression and bipolar disorder [3,7]. In addition to clozapine, modern drugs of interest include aripiprazole, asenapine, brexiprazole, cariprazine, iloperidone, lurasidone, olanzapine, paliperidone, quetiapine, risperidone, and ziprasidone. This background encouraged the present, systematic overview of research pertaining to suicidal risks in relation to treatment with atypical antipsychotic agents. Given the relatively early and underdeveloped state of this area of inquiry, we viewed our task as gathering and critically appraising the available research relevant to the topic, with the aim of formulating a hypothesis to be tested with further research.

## 2. Results

### 2.1. Antipsychotic Drugs in Schizophrenia

Suicide is a major cause of death among patients diagnosed with schizophrenia, accounting for 5%–13% of deaths [8,9]. Risk of suicide appears to be greater among psychotic disorder patients who are younger, male, never married, with evidence of current or previous depression or substance abuse, as well as previous suicide attempts, and who have had relatively good premorbid functioning [8,10,11]. Evidence that older, typical neuroleptic drugs, alone or with antidepressants added, alter suicidal risks in schizophrenia patients, either favorably or unfavorably, is limited and inconsistent [8,12,13,14]. Risk of mortality due to natural causes, including sudden death presumably related to cardiac dysfunction, may be increased with older neuroleptics as well as with modern atypical or second-generation antipsychotics (SGAs) [15,16]. Compelling evidence for the protective or antisuicidal effects of older neuroleptics is lacking, and they have effects that might increase suicidal risk. Notably, some antipsychotics, including clozapine and haloperidol as well as other older and modern antipsychotics, may increase the risk of depression [17,18,19]. In addition, restless agitation (akathisia) is routinely encountered with older antipsychotics, but also with modern atypical agents, and may contribute to the risk of agitated and potentially violent or suicidal behavior [12,20,21].

### 2.2. Antipsychotic Drugs in Bipolar Disorder

Among bipolar disorder patients, in addition to episodes considered as mainly depressive, manic, or hypomanic, both manic and depressive symptoms can occur concurrently or in rapid alternation in mixed states [22,23,24]. Complex mixed bipolar states of mood and behavior encourage especially close clinical evaluation and treatment selection. They are both a strong risk factor for suicidal behavior and a plausible target for treatment with antipsychotic drugs. Relatively narrowly defined mixed states of DSM-IV that simultaneously meet the diagnostic criteria for both mania and major depression have suicidal risks that are even greater than the high rates associated with bipolar disorder in general, with similar risks in types I and II bipolar disorder [25,26,27]. Such patients also have an unfavorable long-term general prognosis [28,29,30,31,32,33]. It remains to be clarified to what extent such poor outcomes may be associated with recently broadened diagnostic criteria for mixed elements of depression and mania as proposed in DSM-5 [22]. In addition, the potential value of antipsychotics in the treatment of such broadly defined and quite prevalent bipolar states remains to be tested [33]. Furthermore, across the complex range of bipolar mixed states, common clinical elements include depressed mood, sleeplessness, pessimism, agitation, anxiety, irritability, or anger, in states of very unpleasant and distressing dysphoria—all of which can contribute to the risk of suicide [24,34]. Relief of dysphoria is a particularly critical therapeutic target. Antipsychotic drugs may contribute to attenuating such suffering, and atypical agents (SGAs) are being used increasingly, empirically, in the clinical management of bipolar disorder patients, as is reviewed below.

### 2.3. Clozapine

Evidence that clozapine has important effects in terms of reduction of suicidal risk and perhaps all-cause mortality in psychotic disorder patients is increasingly secure. Much less has been done to test for such effects in mood disorder patients, although some encouraging evidence is emerging [2]. Briefly, previous studies of the effects of clozapine on suicidal risk date to the early 1990s. Based on a review of over 400 schizophrenia patients, Meltzer and Okayli [35] suggested that clozapine might reduce suicidal risk more than other antipsychotics. A significantly lower rate of all-cause mortality was found among patients who continued vs. discontinued long-term treatment with clozapine [36]. Reductions of suicidal risk were estimated preliminarily to be as high as 85% [37]. These findings encouraged the design of the remarkable International Suicide Prevention Trial (InterSePT) [38].

This landmark trial randomized schizophrenia patients (not necessarily treatment-resistant) selected for having a high risk of suicide, to treatment with clozapine vs. olanzapine. Suicide-related behavior (attempts, interventions) was significantly less among patients treated with clozapine (Hazard Ratio [HR] = 0.76; 95% CI: 0.58–0.97; *p* = 0.03) [38]. With clozapine there were 38% fewer suicide attempts than with olanzapine and 23% fewer hospitalizations for emerging suicidal risk (both *p* < 0.05). Suicide occurred at similarly low rates with both treatments, and was not altered during treatment with clozapine in this [38] or an earlier study [39]. Without additional controls in the InterSePT trial [38], it was not possible to rule out possible benefits associated with olanzapine, even if they were less than those seen for clozapine. In another randomized, rater-blinded study, clozapine was again associated significantly with 22%–25% fewer suicide-related behavioral outcomes than olanzapine among schizophrenia or schizoaffective disorder patients with recent suicide attempts [40].

A meta-analysis based mainly on the findings of six studies pertaining to treatment with clozapine supported a significant overall beneficial effect on suicidal behavior, with a highly significant (*p* < 0.0001), 3.3-fold [CI: 1.7–5.7] lower pooled risk of suicidal behaviors with clozapine compared to alternative treatments [41]. For completed suicides, the pooled risk of completed suicide was 2.9 times [CI: 1.5–5.7] lower with clozapine treatment (*p* = 0.002) [41].

Whether these substantial antisuicidal benefits are associated with clozapine treatment has remained uncertain. One proposal is that a reduction in impulsiveness and aggression rather than depressive symptoms may be important [42]. Another possibility is that closer clinical supervision associated with the use of potentially toxic clozapine (and perhaps also lithium) may contribute to reducing suicidal risk. However, the InterSePT trial [38] balanced clinical contacts with both clozapine and olanzapine. Of considerable historical importance, the FDA granted regulatory approval for the antisuicidal benefit of clozapine in 2003, marking it the first and only treatment of any kind with such regulatory recognition.

Subsequently, Modestin et al. [43], in a retrospective analysis of patients treated with clozapine, found significantly less suicidal behavior among schizophrenia patients treated with clozapine vs. other treatments. Another study concluded that clozapine and olanzapine were both associated with a reduced risk of suicide, attempts, and hospitalization for emerging suicidal risk [44]. In this study of 26,046 schizophrenia patients, the risk of death by suicide (Odds Ratio [OR] = 0.45 [CI: 0.20–0.98]) and risk of attempts (OR = 0.44 [0.28–0.70]) were both significantly lower with clozapine; the benefits were nearly doubled with clozapine compared to haloperidol or olanzapine [44]. Also, a retrospective analysis of findings based on the treatment of 100 psychotic disorder patients found that clozapine and risperidone were associated with more than six-fold fewer suicide attempts compared to treatment with typical neuroleptics (OR = 6.5, *p* < 0.05) [45]. Another recent study also found that patients treated with clozapine had a significantly lower rate of suicide attempts than with olanzapine [46]. In a study that compared several first- vs. second-generation antipsychotics, clozapine was the only second-generation agent associated with lower rates of both suicide and all-cause mortality, whereas other modern agents were associated selectively with less mortality not due to suicide than was found with typical neuroleptics [44].

### 2.4. Olanzapine

That the SGA olanzapine also may have antisuicidal effects was suggested in the 1990s, based on comparing outcomes with olanzapine vs. haloperidol; olanzapine was also associated with other superior clinical benefits, including rating of overall quality of life [47]. Another early trial involving 339 psychotic disorder subjects (diagnosed with schizophrenia, schizoaffective, or schizophreniform disorder) found that both olanzapine and risperidone were safe and effective for the management of psychotic symptoms, but that patients treated with olanzapine had a seven-fold lower risk of suicidal behaviors than those given risperidone (0.6% vs. 4.2%; *p* = 0.03) [48]. In addition, data from premarketing controlled trials indicated a 2.3-fold lower overall rate of suicide attempts in psychotic disorder patients treated with olanzapine vs. haloperidol [49]. In a more recent trial involving 378 subjects who had attempted suicide, there was evidence of the protective effect of several atypical antipsychotic agents, mainly olanzapine and risperidone (OR = 3.5 [CI: 2.4–5.3]; *p* < 0.001) [50].

A meta-analysis of an FDA database of controlled trials found that rates of suicide and attempts (as incidentally reported adverse outcomes) did not differ significantly between drug- and placebo-treated patients among drugs that included olanzapine, risperidone, and quetiapine [51]. However, the trials were not designed to evaluate suicidal behavior as an explicit outcome, did not include assessment methods specific for such risks, and did not clearly match for exposure times across trial-arms.

Also of interest is the combination of olanzapine with the antidepressant fluoxetine. This combination has shown particular efficacy in bipolar depression, and is effective in bipolar mixed states as well as in juveniles with suspected bipolar depression [3,52,53]. These properties suggest that it may be a candidate for having favorable effect on suicidal risk, although we found no reports of studies evaluating suicidal risk specifically with olanzapine plus fluoxetine in any psychiatric disorder.

Finally, it has been reported that discontinuing antipsychotic drug treatment, including olanzapine or risperidone, can be followed by an increase in suicide attempt rates [54]. However, it is not clear whether these changes represented a return to an untreated state or an adverse effect of drug discontinuation itself as an iatrogenic stressor [55].

### 2.5. Quetiapine

Quetiapine has demonstrated some potential for reducing suicidal risk that parallels its established efficacy not only in schizophrenia and mania, but also its emerging efficacy in bipolar and perhaps in unipolar major depression [56,57,58,59,60]. Up to the year 2000, more than 10,000 patient-years of testing were represented in controlled trials of quetiapine [61].

In one of the first randomized, controlled trials for the treatment of bipolar depression, patients randomized to quetiapine vs. a placebo reported greater reductions on the suicide item-10 of the Montgomery-Åsberg Depression Rating Scale (MADRS) [62]. In addition, in a sample of 802 patients with bipolar I or II disorder randomly assigned to quetiapine (300 or 600 mg/day), lithium carbonate (600–1800 mg/day), or a placebo, improvements of bipolar depression were greater with quetiapine than with lithium, with no evidence of a dose effect [63]. Interestingly, improvement in suicidal thoughts was associated with improvements in insomnia and inner tension. In a similar trial, quetiapine (the same dose) was compared with the serotonin-reuptake inhibitor antidepressant paroxetine (20 mg/day) and with a placebo among 740 depressed bipolar I or II disorder subjects; there were significant reductions in depression symptom scores on two standard rating scales (MADRS and Hamilton Depression Rating Scale [HDRS]) associated with quetiapine, but little effect of paroxetine, although the design was not optimized to test for the effects of the antidepressant [64]. These improvements, again, were associated with a decrease in suicidal thoughts as well as in insomnia and inner tension. Another study concluded that quetiapine (at 400–800 mg/day), as well as lithium or valproate, was effective in reducing symptomatic expression of depression and suicidal ideation in a large sample of 1953 bipolar disorder patients treated and followed for at least nine months [65].

Other studies have tested for both antidepressive and possible antisuicidal effects of quetiapine in patients with unipolar as well as bipolar depression [66,67]. In one trial, 612 subjects diagnosed with unipolar major depression were randomized to treatment with quetiapine (150 or 300 mg/day), the antidepressant duloxetine (60 mg/day), or a placebo [66]. Reduction in MADRS depression scores was greater with both doses of quetiapine than with duloxetine or placebo, and included improvements in the suicidal item-10 of the ratings, as well as in insomnia. In another trial, quetiapine (150 or 300 mg/day) was more effective than the placebo in reducing MADRS depression ratings at as early as one week [68]. In yet another trial with 310 depressed subjects, quetiapine (150 mg/day) was more effective than the placebo in treating depressive symptoms, showed some benefit within one day, and again yielded reduced scores on suicidal item-10 of the MADRS depression rating scale [69]. Even unipolar major depressive disorder patients who had not responded adequately to seemingly adequate trials of antidepressant treatment showed improvement within one week after quetiapine (300 mg/day) was added [70].

The effects of quetiapine on suicidal risk were investigated more directly in a study that pooled data from 1766 subjects from six randomized trials in which quetiapine (50–300 mg/day) was either the primary treatment or added to other treatments for unipolar major depression, and compared with a placebo [71]. Findings included rates of suicidal behavior of 1.1% at 50 mg/day of quetiapine compared to 0.7% at higher doses, but without statistical separation from placebo controls in the short term, and a small difference between the placebo and quetiapine (0.5% vs. 0.3% risk) with long-term continuation. There was no evidence that scores on the MADRS suicidal item-10 worsened with quetiapine treatment.

Limitations of the preceding findings arising from controlled treatment trials for quetiapine include a lack of evidence of the effects on suicidal behavior, and study designs that were not aimed explicitly at evaluating suicidal risk. Moreover, individual items on rating scales are not intended for separate analysis, and their scoring can be influenced by an overall improvement in depression ratings. It is also not clear to what extent the potential benefits regarding suicidal risk may be due to improvement of depressed mood or of anxiety, agitation, and insomnia, which appear very early and are likely to be associated with suicidal risk [26,72,73,74]. Despite these shortcomings, the observations just summarized are at least suggestive of possible antisuicidal effects of this atypical antipsychotic, which deserve further and more specific study in patients diagnosed with schizophrenia, bipolar disorder, and major depressive disorder.

### 2.6. Ziprasidone

Reported studies involving ziprasidone appear to be limited to indirect assessments related to suicidal risk, notably by documenting beneficial effects on known risk factors for suicide in persons diagnosed with schizophrenia, including anxiety, depressive symptoms, or impulsive behaviors [57,75], as well perhaps as improvements in cognition, social functioning, and quality of life [76,77]. The difficulty of evaluating suicidal risk more explicitly is illustrated by several trials in which suicidal behaviors were rarely encountered among subjects randomized either to ziprasidone (in doses up to 160 mg/day) or to a placebo [14,78,79,80]. Ziprasidone was included among other atypical antipsychotics in a case-control study of 4000 psychotic disorder patients in Sweden, in which treatment with atypical antipsychotic drugs (including clozapine, olanzapine, and risperidone as well as ziprasidone) yielded fewer completed suicides than alternative treatments. Risk of suicide was reduced by 71% with this class of second-generation antipsychotic drugs overall (OR = 0.29 [CI: 0.09–0.97]), and even slightly more with clozapine-treated cases removed from analysis (OR = 0.23 [CI: 0.06–0.89]). Finally, a pooled analysis of findings from 22 controlled trials of ziprasidone involving 5123 psychotic disorder subjects found that risks of suicidal “events” (ideation or attempts but no suicides) did not differ significantly between subjects randomized to treatment with ziprasidone or a placebo (relative risk [RR] = 0.67 [CI: 0.21–2.20]) [79]. Overall, these findings appear to support the general hypothesis that atypical antipsychotic drugs may have beneficial effects on factors associated with suicidal risk, but that ziprasidone specifically requires further, more focused investigation.

### 2.7. Aripiprazole

Evidence about other atypical antipsychotic drugs that might be pertinent to effects on suicidal risk remains very limited. That aripiprazole has regulatory approved for the acute and maintenance treatment of manic and mixed episodes of bipolar I disorder suggests that some risk factors for suicide might be modified by this agent, including improved social interactions and general quality of life, in addition to antipsychotic and calming effects [57,81]. However, aripiprazole has also been associated with excessive arousal, anxiety, and akathisia, which might increase suicidal risk, as has been suggested in case reports [82,83]. However, a review of clinical observations in a total of 20,489 psychotic disorder patients found rates of suicidal events associated with aripiprazole [22] to be somewhat lower than with quetiapine [33] or ziprasidone (49 per 1000 person-years), with a not significantly different overall rate of such events compared with other antipsychotics after adjustment for the effects of diagnosis, illness severity, and other treatments (HR = 0.78 [CI: 0.48–1.30]) [80].

The effects of aripiprazole in major depressive disorder have also been considered [83]. In data pooled from two trials involving use of aripiprazole (2–20 mg/day) as an adjunctive treatment in 737 depressed subjects, there were slightly fewer reports of suicidal ideation with this atypical antipsychotic, as well as significantly lower scores on suicidal item-10 of MDRS depression ratings. These findings encourage further study of this agent with respect to suicidal risk.

### 2.8. Asenapine

This atypical antipsychotic has regulatory approval for short-term treatment of schizophrenia and for manic or mixed episodes in bipolar I disorder [84,85]. Post hoc analysis of data pooled from two randomized, controlled trials of asenapine in major depressive disorders with hypomanic features supported its superiority over the placebo against both depressive and hypomanic symptoms, but did not address suicidal risk specifically [86]. Other studies also support the value of asenapine in treating both manic and depressive features of bipolar disorder, especially in mixed states [87,88]. Such effects might be expected to reduce suicidal risk, but asenapine requires further study to test for its potential benefits against suicidal risk specifically.

### 2.9. Other Atypical Antipsychotics

Finally, we found no information pertaining specifically to suicidal risk during treatment with other recently introduced atypical antipsychotic drugs, including cariprazine, iloperidone, lurasidone, and paliperidone.

### 2.10. Summary of Findings

Based on the findings arising from the studies just considered, we found that 19 reports provided 24 tests of treatment with specific, second-generation antipsychotic drugs including aripiprazole, clozapine, olanzapine, quetiapine, and ziprasidone vs. a comparison condition (Table 1) [4,5,38,43,44,46,48,49,50,62,63,64,65,66,70,71,79,87,89]. The total number of subjects involved was nearly 87,000 (Table 1). The research methods employed vary, but are based on randomized, controlled designs in 13/24 comparisons, and assessment of effects associated with particular treatments in large, national clinical databases in another 8/24 comparisons. Nevertheless, almost all of these studies involved more or less incidental reporting of suicidal behavior, essentially as an indication of an “adverse effect” rather than on explicit assessments of suicidal behavior with adequately tested methods, such as those employed in the InterSePT trial of clozapine vs. olanzapine in suicidal patients with chronic psychotic disorders [38].

## 3. Discussion

The preceding overview was intended to evaluate the hypothesis that treatment with some modern antipsychotic drugs may tend to limit the risk of suicidal behaviors. The findings just summarized indicate that several atypical or second-generation antipsychotics may possess antisuicidal properties, especially when compared with older neuroleptics. The support for such effects is particularly strong for clozapine, and also is encouraging for olanzapine, quetiapine, ziprasidone, aripiprazole, and asenapine—more or less in that order. The mechanisms by which such effects might occur are not clear. It is not known whether they represent extensions of their general antipsychotic efficacy or reported benefits in mood disorders, including in mania, mixed manic-depressive states, and perhaps in depression [7]. An attractive feature of antipsychotic agents is that the beneficial effects of some of these agents on depression, including in bipolar disorder, present little risk of inducing agitation or mixed manic-depressive states with an associated increase of suicidal risk. However, the risk of akathisia with some modern antipsychotic drugs is of concern for potential worsening of suicidal risks, in addition to the well-known adverse neurological, cardiovascular, and metabolic effects of antipsychotic drugs [3].

In general, the effects of most atypical antipsychotic drugs in reducing suicidal risk have not been adequately tested. An exception is clozapine, a highly effective though potentially toxic antipsychotic drug with established antisuicidal effects in psychotic disorder patients and some preliminary suggestions of such benefits in mood disorder patients as well [12,38,41,90,91]. It remains to be seen how the recently broadened criteria for mixed manic-depressive states [22] will affect indications for antipsychotic drugs and, in turn, interact with suicidal behaviors and their treatment or prevention. Useful information may be forthcoming as more therapeutic trials include explicit assessments of suicidal behaviors rather than relying on potentially misleading ratings of suicidal risk in single items of depression rating scales or on the passive and incidental reporting of suicidal behavior as adverse events [92].

### Limitations

Among the studies cited in this hypothesis-generating overview, subjects and methods varied, and not all provided quantitative assessment of suicide-related behavioral events, so that a formal meta-analysis was not feasible. Also, given the present emphasis on findings that might support the hypothesis that modern antipsychotic medicines may tend to limit suicidal risks, there may well be bias in the findings selected for discussion as well as in the reported findings themselves.

## 4. Methods

We used computerized literature searching to identify reports in English appearing between 1980 and June 2016, of studies related to relationships of antipsychotic agents to risk of suicidal behavior. Relevant reports were sought through the MEDLINE/PubMed^®^, PsychINFO^®^, and EMBASE^®^ literature databases, using the search terms “antipsychotic”, “attempt”, and “suicide”. We also hand-searched bibliographies of screened reports for additional references. Initial screening identified a total of 1306 reports; a review of their abstracts reduced the number of potentially relevant reports to 82, and a final review of full texts yielded 19 reports involving 24 comparisons with findings that could be used in the present review, based on the consensus of at least two authors, plus 73 reports with additional, relevant information. Target outcomes were suicides and attempts, with secondary consideration of suicidal ideation, all as specified in the studies cited below.

## 5. Conclusions

The support for the beneficial, risk-reducing effects of modern antipsychotic drugs is particularly strong for clozapine—to date, the only treatment with regulatory approval for reducing suicidal risk, at least in schizophrenia patients. In addition, we found support for the hypothesis that olanzapine, quetiapine, ziprasidone, aripiprazole, and asenapine may all have suicidal risk-reducing effects, including among persons with major affective or psychotic disorders. These leads require additional study, ideally in ethical and feasible controlled trials that compare plausible alternative active treatments. Such trials should be based on explicit assessment of suicide-related outcomes—ideally, behaviors rather than subjective suicidal thoughts, and with due consideration of the added risks that may arise with akathisia.

## Figures and Tables

**Table 1:** Summary of reports on modern antipsychotic drugs and outcomes related to suicidal risk or depression.

Study	Subjects (*n*)	Diagnosis	Study Design	Comparison Treatment	Outcome Measure	Risk Reduction	Comments
*Aripiprazole*							
Weisler et al. 2011 [89]	737	MDD	Pooled RCTs	Placebo	Suicidal ideation	–––	Ariprazole effective
Ringbäck-Weitoft et al. 2014 [44]	720	Sz	National database	APs, SGAs	Suicide	OR: 0.21 [0.05–0.94]	Ariprazole effective
*Asenapine*							
Azorin et al. 2013 [87]	295	BD-I	Pooled RCTs	Olanzapine	Depression	–––	Asenapine effective vs. depression & suicidal ideation
*Clozapine*							
Meltzer et al. 2003 [38]	980	Sz	RCT	Olanzapine	Suicidal behavior	HR: 0.76 [0.58–0.97]	Clozapine superior
Modestin et al. 2005 [43]	94	Sz	Chart review	±ADs	Suicidal behavior	–––	Clozapine effective ± antidepressants
Haukka et al. 2008 [4]	504	Sz	National database	No treatment	Suicide	HR: 0.74 [0.35–1.57]	Clozapine reduced mortality
Tiihonen et al. 2009 [5]	32,000	Sz	National database	Perphenazine	Suicide	HR: 0.34 [0·20–0·57]	Clozapine reduced mortality
Ringbäck-Weitoft et al. 2014 [44]	2138	Sz	National database	APs, SGAs	Suicidal behavior	OR: 0.45 [0.20-0.98]	Clozapine more effective than haloperidol
Thomas et al. 2015 [16]	–––	Sz	Open trial	Olanzapine	Suicide attempt	–––	Clozapine more effective than olanzapine vs. suicide attempts
*Olanzapine*							
Tollefson et al. 1997 [49]	1996	Sz	RCT	Haloperidol	Negative symptoms	–––	Olanzapine more effective vs. suicidal ideation & negative symptoms
Tran et al. 1997 [48]	339	Sz	RCT	Risperidone	Negative symptoms	–––	Olanzapine more effective vs. suicidal ideation & negative symptoms
Barak et al. 2004 [50]	756	Sz	Case-control chart review	Risperidone or Neuroleptics	Suicide attempt	OR: 0.57 [0.30–0.83]	Olanzapine & risperidone more effective than neuroleptics
Haukka et al. 2008 [4]	394	Sz	National database	No treatment	Suicide attempt	HR: 1.37 [0.87–2.14]	Olanzapine effective
Tiihonen et al. 2009 [5]	25,130	Sz	National database	Perphenazine	Suicide	HR: 0.94 [0·61–1·45]	Olanzapine more effective
Ringbäck-Weitoft et al. 2014 [44]	3747	Sz	National database	APs, SGAs	Suicide attempt	OR: 0.44 [0.28-0.70]	Olanzapine more effective
*Quetiapine*							
Calabrese et al. 2005 [62]	511	BD-I+II	RCT	Placebo	Suicidal thoughts	–––	Quetiapine more effective
Cutler et al. 2009 [66]	612	MDD	RCT	Placebo	Suicidal thoughts	–––	Quetiapine (150–300 mg/day) effective
Suppes et al. 2009 [65]	1953	BD-I	RCT	Placebo	Depression	–––	Quetiapine + lithium or + divalproex reduced new depression 67%
Tiihonen et al. 2009 [5]	5360	Sz	National database	Perphenazine	Suicide	OR: 1.58 [0.89–2.79]	Quetiapine not effective
El-Khalili et al. 2010 [70]	446	BD-I+II	RCT	Placebo	Suicidal thoughts	–––	Quetiapine effective for suicidal thoughts, tension, insomnia
McElroy et al. 2010 [64]	740	BD-I+II	RCT	Placebo	Depression	–––	Quetiapine more effective vs. suicidal ideation & depression
Young et al. 2010 [63]	802	BD-I+II	RCT	Placebo	Depression	–––	Quetiapine effective vs. suicidal thoughts, tension, insomnia
Weisler et al. 2014 [71]	1766	MDD	Pooled RCTs	Placebo	Suicidal behavior	–––	No emergent suicidal behavior (drug or placebo)
*Ziprasidone*							
Karayal et al. 2011 [79]	5123	BD-I+II	Pooled RCTs	Placebo	Suicidal behavior	–––	No emergent suicidal behavior (drug or placebo)

Abbreviations: AD, antidepressant; AP, antipsychotic; BD, bipolar disorder (type I or II); HR, Hazard Ratio; MDD, major depressive disorder; OR, Odds Ratio; SGA, second-generation antipsychotic; Sz, schizophrenia; RCT: randomized controlled trial; –––, no information. The 24 comparisons summarized here arise from 19 reports [4,5,38,43,44,46,48,49,50,62,63,64,65,66,70,71,79,87,89], and include over 86,896 subjects.
